# *Mardivirus* Infection and Persistence in Feathers of a Chicken Model Harboring a Local Autoimmune Response

**DOI:** 10.3390/microorganisms8101613

**Published:** 2020-10-20

**Authors:** Gisela F. Erf, Gilles Le Pape, Sylvie Rémy, Caroline Denesvre

**Affiliations:** 1 Department of Poultry Science, University of Arkansas, Fayetteville, AR 72701, USA; gferf@uark.edu; 2 Anastats, 14 rue de la Bretonnerie, 37000 Tours, France; lepape.gilles@anastats.fr; 3 ISP, INRAE, Université Tours, 37380 Nouzilly, France; sylvie.remy-delaunay@inrae.fr

**Keywords:** herpesvirus of turkey, Marek’s disease vaccine, Smyth line, vitiligo, feathers, viral persistence

## Abstract

Herpesvirus of turkey (HVT) is commonly used as a vaccine to protect chickens against Marek’s disease. Following vaccination, HVT infects feathers where it can be detected in all chicken lines examined. Unlike the parental Brown line (BL), Smyth line (SL) chickens develop vitiligo, due to autoimmune destruction of melanocytes in feathers. Previous reports showed a strong inflammatory response in Smyth chickens’ feathers at vitiligo onset, that subsided once melanocytes were destroyed, and depigmentation was complete. Here, we questioned whether the local autoimmune response in the Smyth model influences HVT infection and persistence in feathers. For this, one-day-old SL and BL chickens were vaccinated with Newcastle disease (rHVT-ND). Vitiligo was scored and HVT loads in pigmented and non-pigmented growing feathers were quantified regularly over 20 weeks. Chickens of both lines showed moderate HVT loads in feathers. At the onset of active vitiligo, the HVT load was significantly higher in SL compared to BL feathers. However, no difference in HVT loads was noticed between pigmented and non-pigmented feathers from SL chickens. Therefore, surprisingly, the inflammatory response in feathers of SL chickens did not inhibit HVT infection and persistence, but on the contrary, temporarily promoted HVT infection in feathers.

## 1. Introduction

The *Meleagrid herpesvirus* (MeHV), commonly named herpesvirus of turkey (HVT), is extensively used in the poultry industry worldwide to protect against Marek’s disease (MD) [1,2]. Indeed, this non-pathogenic virus belonging to the *Mardivirus* genus in the *Alphaherpesvirus* sub-family is genetically and serologically related to Marek’s disease virus (MDV) or *gallid herpesvirus type 2*. For the past two decades, this virus is also increasingly used as a platform vector to develop recombinant vaccines against other major viral chicken diseases, like infectious bursal disease and Newcastle disease (ND) [2,3,4,5,6]. All HVT vaccines, recombinant or not, are live vaccines. In chickens, HVT, like the oncogenic MDV, has a tropism for lymphoid tissues as well as the skin (reviewed in [7,8]). HVT appears to establish latency mostly in spleen cells, in which the HVT genome integrates into host chromosomes [9]. In the skin, HVT infection persists for weeks in feathers [10]. In this tissue, HVT infection is considered productive like MDV, based on viral shedding, evidenced by the presence of virus genome on the surface of the skin, in dander, and poultry dust [11,12,13]. In chickens, HVT persistence in feathers is not a problem because of its apathogenic phenotype and its poor horizontal transmissibility [14,15]. In contrast, viral persistence in feathers and dander is a major problem with pathogenic MDV. With MDV’s ability to persist for weeks in these materials, it constitutes a unique vehicle for horizontal MDV transmission [16]. In particular, in commercial farm settings, where complete dust removal is difficult, pathogenic MDV may durably contaminate the environment. Therefore, an understanding of *Mardivirus* persistence in feathers and how to eradicate it are major issues for poultry health, sustainability of poultry production, and for more effective control of environmental contamination by pathogens. HVT is a good virus model to identify factors that modulate *Mardivirus* infection and persistence in feathers.

The Smyth line (SL) of chickens is an interesting chicken model to examine *Mardivirus* infection in feathers. Chickens from this line spontaneously develop vitiligo-like loss of feather pigmentation due to melanocyte-specific autoimmune responses that result in the apoptotic death of epidermal melanocytes in growing feathers (GFs) [17,18,19]. Like other spontaneously occurring autoimmune diseases, SL vitiligo (SLV) is a non-communicable, multifactorial disorder, involving genetic, environmental, and immunological factors in disease expression. In SLV, genetic susceptibility appears to involve melanocyte abnormalities that are, however, not sufficient for disease expression without a competent immune system [20]. Melanocyte-specific cell-mediated immunity as well as autoantibodies are present in chickens that developed SLV [21,22]. Melanocyte loss in GFs is associated with extensive T and B cell infiltration in feathers locally. CD4+ T cells dominate the autoimmune response prior to and at onset of SLV, whereas progression of vitiligo and melanocyte loss are associated with sustained presence of CD8+ T cells, which are also found in close association with apoptotic melanocytes in the barb ridge. In addition to interferon-γ (IFN-γ), the cytokine signature of the autoimmune response includes IL-6, IL-8 (aka CXCL8), IL-21, and IL-10 [23]. Therefore, during the vitiligo process, feathers show a strong local pro-inflammatory cell-mediated immune response environment [17,23,24,25]. Live virus vaccination with HVT (and other MDV serotypes) at hatch was identified as an environmental trigger of SLV expression. The spontaneous incidence of SLV in an HVT-vaccinated SL population is 70–95%, whereas, without vaccination, the incidence is <20% [26]. In addition to the *Mardivirus* connection in SLV development, the availability of a parental control, namely the Brown line (BL) from which the SL originates, further underlines the suitability of this animal model for exploring HVT infection and persistence in GFs. Vitiligo susceptibility is maintained in BL chickens, however, less than 2% of the BL population exhibit pigmentation loss even with HVT vaccination at hatch [19,27]. SL and parental BL chickens are from the same genetic background and MHC matched (*B* locus, *B*^101/101^), and hence are expected to mount comparable immune responses to HVT as they do when immunized with sheep red blood cells [28].

The vitiligo-prone SL and parental BL chickens constitute an excellent opportunity to examine whether vitiligo development and the associated local T cell-mediated inflammatory response in GFs can reduce HVT infection of feathers and/or modulate its replication kinetics as well as persistence. Using a recombinant HVT-ND vaccine, HVT infection and persistence in GFs from vitiligo-prone SL and normally pigmented BL chickens were monitored every 3 weeks starting at 2 weeks of age (2 weeks post-vaccination) and throughout the 20 weeks when SLV develops. Additionally, levels of NDV antibodies were examined when the chicks were 13 weeks of age to confirm immunogenicity and success of the vaccination.

## 2. Materials and Methods

### 2.1. Experimental Chickens and In Vivo Study Design

The University of Arkansas Institutional Animal Care and Use Committee (IACUC) approved all protocols and procedures involving animals used in this trial (IACUC Protocols # 15015 and #18049, 7/2014-7/2017 and 6/2017-2/2020 respectively). Chicks used in this study were offspring from the annual breeder replacement pedigree hatch of SL and BL populations maintained by G. F. Erf at the Arkansas Agricultural Experiment Station Poultry Farm at the University of Arkansas, Fayetteville. At hatch, all chicks were individually tagged for identification and vaccinated (s.c.) with a single dose of live rHVT-ND vaccine (Vectormune^®^ HVT-ND, CEVA Animal Health, LLC, Lenexa, KS serial number 372-1004, 4000 pfu-dose) as recommended by the manufacturer. Note: All chicks came from dams that were vaccinated with live HVT (Fort Dodge Animal Health, Fort Dodge, IA); hence, chicks may have had maternal antibodies to HVT, but not to NDV. Chicks were placed in floor pens and raised using conventional rearing conditions on wood shaving litter with food and water available ad libitum.

### 2.2. Vitiligo Scoring and Feather Sampling

Nine female BL chicks and eleven female SL chicks were used for this study. Note that a 12th SL chicken (SL03, not shown) was negative for NDV antibodies and did not develop SLV; hence, she was considered not HVT-ND vaccinated and excluded from all analyses. Prior to sampling, all chicks were visually scored for signs of depigmentation, indicative of vitiligo onset. Scoring was done by close examination of the most proximal end (newest growth of GFs). Scores were assigned as follows: 1 (all GFs pigmented) to 5 (100% of GFs depigmented—complete amelanosis). In a few cases, feather re-pigmentation was observed and scored “RepigYes”, in contrast to “RepigNo”. Both scoring and GF sampling of the 20 birds (four GFs each from axillary tracts, neck, and rump) began at 2 weeks of age and continued every 3 weeks until the chickens were 20 weeks of age. Week 20 was chosen to stop the experiment because at this age mature plumage is established, only few regenerating feathers are present and SLV development ends because epidermal melanocytes are only present in GFs [18]. For SL chicks with vitiligo scores 2, 3, and 4, with 1 to 29%, 30 to 60%, 60 to 99% of all GFs affected, respectively, pigmented and non-pigmented GFs were sampled, resulting in six to 10 feather samples per bird. For each bird and sample time, collected GFs were flash frozen in liquid nitrogen, and stored at −80 °C until use for DNA isolation. When the chickens were 11 weeks of age, peripheral blood (2 mL) was collected from the wing vein. Blood was used for plasma preparation and titration of antibodies to NDV. After week 20, the experimental birds were returned to the breeder replacement population. After initiation of the production cycle by conventional light stimulation, some of the experimental birds were culled from the population. Those chosen to be kept as part of the breeding population (seven SL and seven BL hens) were euthanized by i.v. injection of sodium pentobarbital (Sigma) at 15.5 months of age as part of the yearly breeder replacement cycle. At time of euthanization, birds were necropsied and spleens harvested for HVT load quantification.

### 2.3. DNA Preparation and Absolute Quantitation of HVT Load by qPCR in Growing Feathers and Spleens

All protocols were previously described [10]. Briefly, feather epithelium and pulp were collected mechanically by pressing and rubbing a scalpel blade on the sheaths of GF tips. DNA was extracted with the QIAamp mini kit (Qiagen). Two absolute qPCRs were performed on each sample with the Taqman technology, one for a HVT sequence and one for the *iNOS* cellular gene. All HVT loads in feathers and spleens were expressed as HVT genome copy number per million cells.

### 2.4. HVT Vaccine Uptake by NDV Antibody Titration

To verify that the birds were correctly vaccinated, NDV antibody titers in the plasma were determined at week 11 post-vaccination by ELISA (ID screen Newcastle Disease indirect kit, IDvet, France). Titrations were generously performed by CEVA BIOMUNE, Lenexa, KS, USA.

### 2.5. Statistical Analysis

The variables are: the chicken line (BL, SL), the date of sampling (from week 2 to week 20, abbreviated as W02 to W20, the vitiligo score, HVT load in feathers (from pigmented feathers “HVT_A”, from non-pigmented feathers “HVT_B”, from all feathers pigmented and non-pigmented feathers “HVT_AB”). Two data points were missing, corresponding to uninterpretable qPCR, and re-imputated (SL2W08HVT_A; SL4W17HVT_B). Re-imputation was made for two data points by replacing the missing data by the mean of the two values that framed the missing data (e.g., the missing value of SL2W08HVT_A was extrapolated from the measures obtained at weeks 5 and 11). Because of small sample sizes and the impossibility to test normality of distributions, only non-parametric tests were used. For HVT loads, all tests were performed after decimal logarithm transformation. Permutation tests were used on numerical values when homogeneity of variances was verified, otherwise on ranks. Comparisons of proportions were performed by Fisher’s exact test. For comparisons of independent samples (e.g., lines at a given date) or dependent samples (e.g., dates for a given line), permutation methods for ANOVA were used, either exact, if made possible by the sample size, or by Monte Carlo approximation. For analysis of variance in the case of mixed models (e.g., several lines at different dates), a non-parametric ANOVA-like test for non-parametric analysis of longitudinal data in factorial experiments using ranks and adjusted *p*-values for pairwise comparisons was used [29,30]. GraphPad Prism version 7 (San Diego, CA, USA) and R software version 3.6.0 (https://www.R-project.org/) were used for plots and computing.

## 3. Results

### 3.1. Vitiligo Development Over Time and Vaccine Uptake

**Figure 1:**
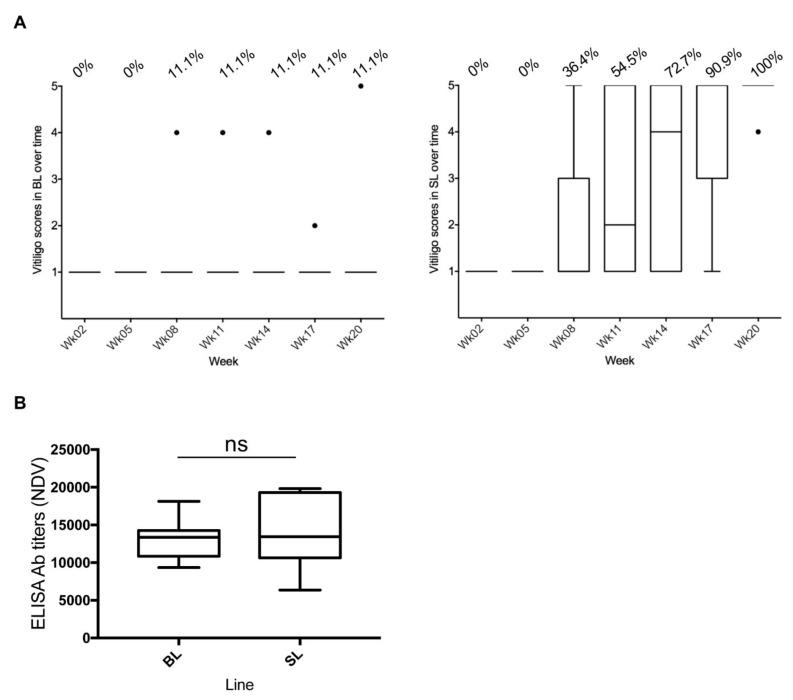
Herpes virus of turkey–Newcastle disease (HVT-ND) vaccination and vitiligo development in Smyth line chickens. All data are shown in Tukey boxes with medians as horizontal bars and interquartile ranges as vertical bars with notches. The black individual dots represent outliers. (**A**) Vitiligo score in Brown line (BL) and Smyth line (SL) chickens over time. A score of 1 corresponds to “no vitiligo” and a score of 5 to “complete vitiligo”. The percentage of birds having developed vitiligo is indicated above the graphs for each time. One hundred percent of SL chickens (*n* = 11) had developed severe vitiligo at week 20, as did one of the BL chickens (*n* = 9). (**B**) HVT-ND uptake. NDV antibody titers in plasma were measured at week 11 by ELISA. All birds had elevated titers, mostly above 10,000, indicating good vaccine uptake and antibody response. ns, non-significant.

Feather depigmentation was observed in week 8 in both lines, with all 11 SL (100%) and 1 BL (BL03; 11.1%) chickens developing severe vitiligo (score 4–5) by 20 weeks (Figure 1A; Table S1). The onset of vitiligo in SL chickens occurred between week 8 and week 17, with 54% of experimental chickens presenting SLV in week 11.

The HVT-ND uptake was verified by measuring NDV antibody titers in plasma in week 11, by ELISA. All 20 chickens (BL and SL) had NDV antibody titers, with 90% above 10,000, indicating effective vaccine uptake and antibody response to the HVT-ND vaccine (Figure 1B). A Mann–Whitney test revealed no difference in NDV antibody titers between the two lines (*p* = 0.6698), indicating that both lines mounted a similar antibody response to HVT-ND vaccine.

### 3.2. HVT Infection and Load in Feathers Collected from SL Compared to BL Chickens

**Figure 2:**
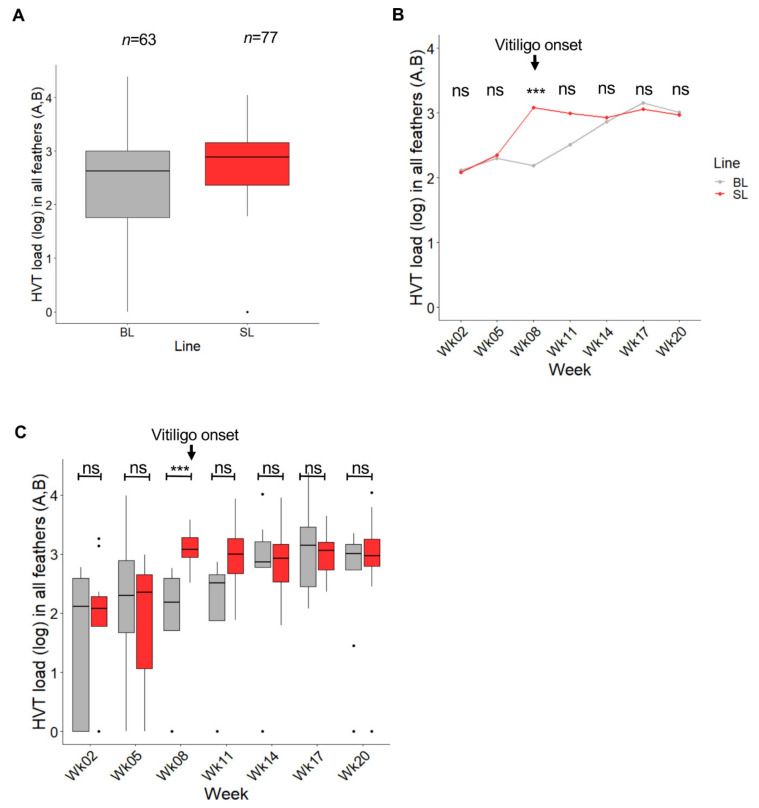
Influence of the chicken line (BL vs. SL) on HVT load in feathers independent of pigmentation. Data are shown in gray for BL and in red for SL. Feathers of all types were considered herein (pigmented or not) and abbreviated feathers (**A**,**B**). The HVT load unit is HVT genome copy number per million cells. (**A**) HVT load across time. Data are shown in Tukey boxes with the log medians visible as thick horizontal bars (2.629 for BL, 2.889 for SL). The HVT loads were moderate in both lines. (**B**) Dynamic of HVT load over time. Each dot corresponds to the median load (log) at a time point. The difference in HVT load was significant at week 8 (adjusted *p*-value < 0.001, ***; Wilcoxon test with a Holm correction for multiple comparison). ns, non-significant. (**C**) HVT load per line over time. Data are shown in Tukey boxes as above. ns, non-significant.

HVT load (genome copy number per million cells) in pigmented and non-pigmented feather samples was determined using qPCR. Note: for birds with vitiligo scores of 2 to 4, both pigmented and non-pigmented GFs were collected. Of 140 expected measures (63 for BL and 77 for SL), all were obtained after two imputations (see Materials and Methods; Table S1). Independent of collection time, HVT load in pigmented and/or non-pigmented feathers was of a moderate level in BL and in SL GFs (median of 425.6 and 774.5, respectively) (Figure 2A). The two boxplots overlapped extensively, and the medians were close, indicating no or marginal differences in HVT load in feathers between the two lines regardless of time post-vaccination. Examination of HVT load at each time point revealed a similar median HVT load (log) for SL and BL at weeks 2 and 5 (Figure 2B). At week 8, the HVT load in SL greatly increased, whereas it remained almost stable in BL. Thereafter, the HVT load increased in BL too, reaching the level of SL at weeks 14 and 17. A Wilcoxon test with a Holm correction for multiple comparisons indicated that the difference between the two lines was significant only at week 8 (adjusted-*p* = 0.00083; SL > BL), the time at which vitiligo was first observed in SL chickens. A boxplot representation of HVT loads in the two lines over time (Figure 2C) indicated that the variability of HVT loads in the first weeks was higher in BL than in SL, with no overlaps between the boxes at week 8 and 11, and tended to become similar thereafter. An ANOVA-like test for mixed model showed a significant interaction between line and week and indicated that the increases in HVT loads over time were different in the two lines (line *p*-value: 0.0998; week *p*-value: 9.00 10-6; line:week interaction *p*-value: 0.0095). Therefore, HVT infection was faster and/or higher in SL than in BL around vitiligo onset.

**Figure 3:**
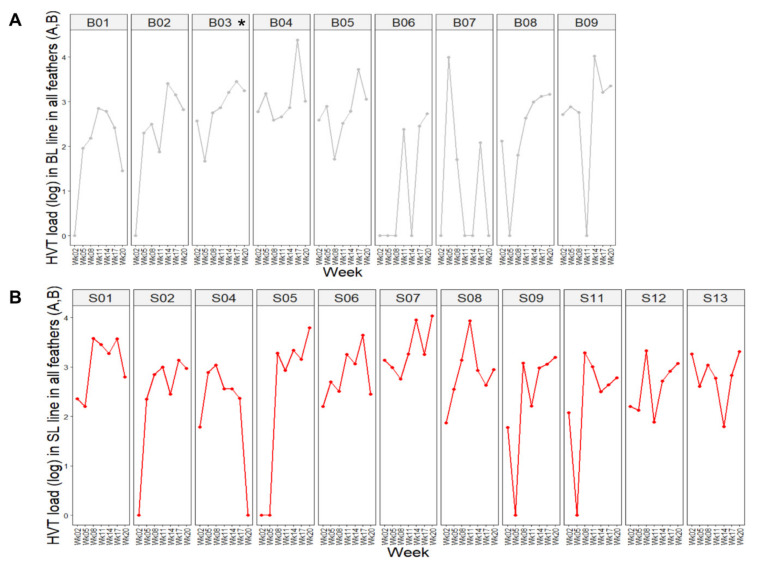
Dynamic of HVT load per subject over time. The HVT load unit is HVT genome copy number per million cells. Each dot corresponds to the HVT load at a time point. (**A**) BL chickens. (**B**) SL chickens. The birds exhibited two major profiles: (**i**) chickens positive at all time points, like B04 or S06; (**ii**) birds showing one or more null-HVT loads, notably after week 2, like B07 and S09. In this figure, BL and SL are abbreviated as B and S, respectively.

Examination of the time course profiles of HVT load in feathers established for each SL and BL chicken revealed two types of profiles (Figure 3): chickens harboring detectable HVT (positive) at all time points in their feathers (33% for BL, 55% for SL), and chickens showing one to four time points with undetectable HVT in their feathers (67% for BL, 46% for SL). A comparison of the proportions for each profile with an exact Fisher’s test showed no differences between the two lines (*p*-value = 0.4059).

### 3.3. Comparison of HVT Loads in Pigmented and Non-Pigmented Feathers from SL Chickens

**Figure 4:**
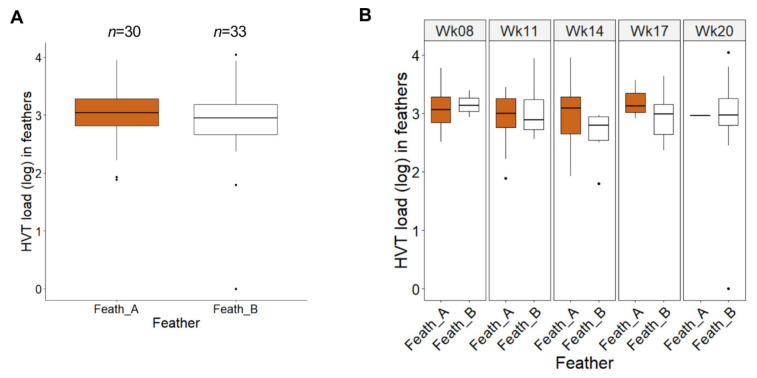
Comparison between HVT load in pigmented and non-pigmented feathers in vitiliginous SL chickens from week 8 to 20. Pigmented feathers (Feath_A in brown); non-pigmented feathers (Feath_B in white). The HVT load unit is HVT genome copy number per million cells. (**A**) HVT loads across time. Data are shown in Tukey boxes with the log medians visible as thick horizontal bars (3.037 for Feath_A, 2.951 for Feath_B). The two boxplots highly overlap indicating no or very low differences in HVT loads between pigmented and non-pigmented feathers in SL. (**B**) HVT loads at individual times. Data are shown in Tukey boxes as above. No significant differences in HVT loads were observed between pigmented (Feath_A) and not pigmented feathers (Feath_B) (all adjusted *p*-value > 0.9; exact Wilcoxon test with a Holm correction for multiple comparisons).

To determine whether active vitiligo in a feather influences HVT replication, we compared HVT loads in all pigmented and non-pigmented SL feathers from week 8 (the time vitiligo was first recorded) to week 20. The feather load medians in both types of feathers were of moderate level and numerically close: 1088 for pigmented feathers (A; *n* = 30); 893 for non-pigmented feathers (B; *n* = 33) (Figure 4A). In addition, the boxplots for pigmented and non-pigmented feathers were widely overlapping. No statistical test was applicable here due to a mix of independent and paired measures (between individuals and time points, respectively), because of the lack of pigmented or non-pigmented GFs on some of the sampling dates and birds. However, the graph suggested no difference in HVT load between pigmented and non-pigmented feathers. Similar results were obtained when comparing the two types of SL feathers over time (Figure 4B). With the amounts of data per week not being constant, a global test for mixed data was not applicable. An exact Wilcoxon test for each week with a Holm adjustment for multiple comparisons revealed no significant differences between the loads in pigmented and non-pigmented feathers at each time in SL chickens (all adjusted *p*-values were above 0.9). Altogether, these results indicate that the stable depigmentation state of feathers had no influence on HVT replication efficacy.

Four birds showed re-pigmentation event(s) of their feathers (BL03, SL01, SL05, and SL11). In all cases, these re-pigmentation events were incomplete (vitiligo score ≥ 2) and transient, as all four birds had stable vitiligo with a score of 5 at week 20. We did not notice any peculiarity for these birds in terms of re-pigmentation events and HVT loads (not shown).

### 3.4. HVT Persistence in the Spleen of the Two Lines at 15.5 Months Post-Vaccination

**Figure 5:**
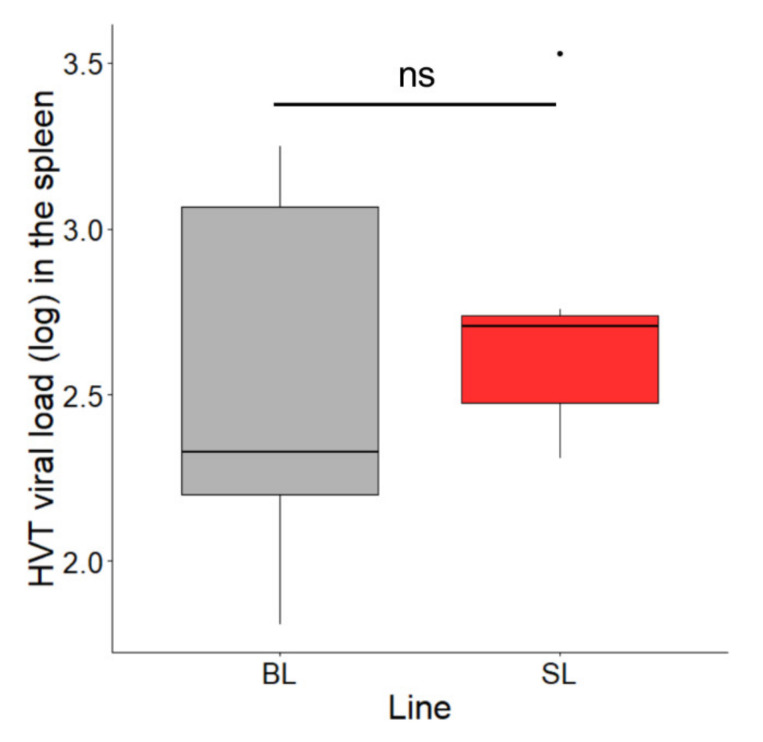
HVT loads in the spleen at 15.5 months of age in SL and BL chickens. Seven birds of each line were analyzed. The HVT load unit is HVT genome copy number per million cells. Data are shown in Tukey boxes with the medians visible as thick horizontal bars (212 for BL, 507 for SL). The difference in HVT load in the spleen was not significant between the two lines (exact *p*-value = 0.7995; permutation test for two independent groups). ns, non-significant.

MD vaccines are known to persist for several weeks in the spleen of vaccinated birds. To determine whether the SL phenotype affects this feature, we analyzed HVT loads in the spleen of seven birds from each line at 15.5 months of age. The median HVT loads were 212 in BL and 507 in SL (Figure 5). A permutation test for two independent groups was applied and showed no significant difference in HVT loads in the spleen between the two lines (exact *p*-value = 0.7995). This result indicates that the genetic defect associated with SLV expression in SL chickens does not influence HVT persistence in the spleen.

## 4. Discussion

Chickens from the vitiligo-prone Smyth line (SL) and the parental Brown line (BL) provide a unique opportunity to examine *Mardivirus* infection and persistence in feathers in the presence and absence of local T cell-mediated immune responses. Specifically, both SL and BL chickens are genetically similar and share the same MHC genotype (*B*^101/101^), however, SL chickens predictably develop a high incidence of autoimmune loss of melanocytes (vitiligo) while BL chickens rarely express this disorder. *Mardivirus* infection and the melanocyte-specific autoimmune response share the same target tissue—the feather. Moreover, expression of SLV is strongly associated with live HVT administration at hatch. In order to better decipher the mutual interactions between HVT and vitiligo development in chickens, HVT infection of feathers in SL chickens was measured through qPCR HVT-specific genome loads over 20 weeks, before and throughout vitiligo development, and compared to HVT infection in the parental BL controls. The HVT loads in feathers of SL and BL were moderate, rarely exceeding 10^4^ HVT genome copies per million cells, with medians of 774 and 425, respectively. These viral loads were in accordance with the ones we reported in feathers in four different lines vaccinated at hatch with the same HVT-ND vaccine [10]. These viral loads were also similar to the ones reported by others in feathers of birds vaccinated with classical HVT vaccines in the first weeks post-vaccination, independent of the presence or absence of maternal antibodies against MDV [11,31]. Altogether, these results indicate that SL and BL harbor similar HVT loads in feathers, that are also not different from those reported for other chicken lines.

We observed some feather samplings with no detectable HVT genomes at some time points. We reported similar results earlier [10]. A chicken has more than 20,000 feathers and DNA was extracted from only two to three feathers at each sampling. There are several explanations possible for undetectable HVT: (i) the host eliminated the infection from the plucked feathers; and/or (ii) only some of the feathers of a bird are infected and uninfected feathers were plucked. The presence of PCR inhibitors was discarded as an explanation, because all samples were amplified with iNOS qPCR. In the absence of any systematic analysis of a large population of feathers from several birds vaccinated with HVT, it is not possible to determine whether an undetectable HVT genome is due to a bias of sampling or reflects what is occurring in all feathers. At week 2, the absence of HVT DNA in feathers of some chickens also might have been due to a slight delay of HVT to reach feathers in comparison to MDV, due to its lower replication level in lymphoid compartments.

It is important to question whether HVT genome copies measured in feathers are associated with HVT replication and/or correspond to the presence of latently infected T cells. In a previous theoretical calculation for a normal chicken, we estimated that 20 copies per million pulp cells may come from latently infected CD4+ T cells [10]. As feather tip material collected contained both feather pulp and epithelium, we can further reduce this number to about 10 copies per million feather cells possibly associated to latently infected CD4+ T cells. This number is negligible compared to the number of copies usually detected in feathers. For SL chickens, this calculation needs to be revised due to the autoimmune response. Shi found that the relative proportion (% area) of CD4+ T cells in longitudinal sections of feather pulp changed over time from 0.61% in samples from non-vitiligo SL chickens, to 2.35% just before (<2 weeks) onset and 4.81% during active vitiligo [23]. Based on the calculation above, in vitiliginous SL, we can expect 38 to 78 copies coming from latently infected CD4+ T cells. Therefore, for vitiliginous SL chickens, we cannot exclude that the presence of latently infected CD4+ T cells may have contributed to the HVT loads. Nevertheless, the higher frequency of CD4+ T cells in SL feathers during vitiligo alone cannot explain the viral loads observed.

We found that SL chickens exhibited higher HVT loads in feathers than BL at 8 weeks post-vaccination, although HVT feather loads were not significantly different in SL compared to BL chickens when examined across all sample times. Week 8 coincides with the first recording of vitiligo that had developed in 36.4% birds between the 5- and 8-week samplings. The age of vitiligo onset with HVT-ND is in accordance with our previous observations of vitiligo development between 6 and 16 weeks of age in SL chickens vaccinated with classical HVT [18]. It has been well established that onset and progression of SLV and active melanocyte loss in growing feathers is proceeded and accompanied by massive infiltration of T cells as well as an increase in pro-inflammatory cytokines, not observed in age-matched normally pigmented SL and BL feathers [23,24,32]. Therefore, we propose three hypotheses to explain this boost in HVT load at 8 weeks in SL feathers: (i) a massive arrival of latently HVT-infected CD4+ T cells in the feathers associated with vitiligo onset, (ii) a positive effect of the inflammatory context on HVT reactivation from latently infected T cells, already present in feathers, (iii) a positive effect of the inflammatory context on HVT replication in keratinocytes. Our previous observation, that following vaccination with HVT at day of hatch, immune cells arrive quicker and reach higher levels in skin and feathers in SL than in BL chickens [18], is in accordance with the first hypothesis. In contrast, the fact that there were no differences between the percentage of birds showing HVT positive feathers at 8 weeks post-infection (100% in SL versus 89% in BL) does not support it, nor does the calculation above. Secondly, melanocyte death in vitiligo is thought to be associated with oxidative stress in the epidermis [19,33,34]. As we demonstrated earlier that genotoxic agents and, less efficiently, oxidants are activators of MDV reactivation in latently infected CD4+ T lymphocytes [35], the second hypothesis is therefore plausible. Note that promotion of HVT reactivation by increased UV exposure due to the absence of melanocytes cannot be evoked here because chickens were raised indoors. Several studies support the third hypothesis. The knock-down of cyclooxygenase 2, an enzyme involved in inflammation, reduces MDV replication [36]. In addition, an enhancement of virus replication through inflammatory mediators was observed for the human cytomegalovirus, another herpesvirus, in human retinal pigment epithelial cells [37]. A comparable process also was observed for viral transmission of the human T cell leukemia virus [38], a retrovirus tightly associated with lymphocytes like MDV and HVT.

Depigmented feathers, corresponding to feathers in which melanocytes were eliminated, had similar HVT loads than pigmented feathers. This result indicates that melanocytes do not play an essential role in HVT infection and persistence in feathers and that melanocytes are not a reservoir for HVT in feathers, as we hypothesized earlier [8].

Based on earlier immunological studies in BL and SL chickens, we wonder here if a high level of IFN-γ locally influences HVT infection/replication in feathers. IFN-γ is a pleiotropic molecule well known to support Th1 response and to activate dendritic cells, macrophages, and NK cells [39]. If antiviral functions of IFN-γ have been depicted, including towards herpesviruses [40,41], the mechanisms remain poorly understood. Herein, we did not observe any reduction of HVT loads and even a tendency to have higher viral loads (notably at 8 weeks) in SL compared to BL feathers. Since the SL birds harbor high IFN-γ transcript and protein levels in their feather pulps 1 week prior and throughout vitiligo development [23,25], our result indirectly indicates that IFN-γ is not associated with reduced HVT replication in feathers. At first sight, these results were unexpected. Indeed, it was shown before that chicken IFN-γ added to CEF infected with MDV or HVT reduces virus replication in vitro [42,43]. It was also reported that intraabdominal administration of chicken IFN-γ every third day to chicks infected with MDV delays tumor formation and death. As viral loads in PBMC and feathers were not measured in this in vivo study, it is difficult to discern whether IFN-γ partially impaired viral replication or only slowed tumor development. Other studies suggest that IFN-γ has little impact on MDV replication in feathers, a result which is more in accordance with ours. Indeed, it was observed that IFN-γ RNA expression was more upregulated in the feather pulps of MD-susceptible P2A birds than in MD-resistant N2A, with P2A having about 100 times higher MDV genome loads in feathers than N2A at 21 days post-infection [44]. Moreover, administration of three recombinant avian adeno-associated viruses expressing a shRNA targeting IFN-γ before MDV infection did not modify MDV loads in feathers [45]. Altogether, our study contributes additional evidence that in the presence of IFN-γ, HVT and MDV infection persists in feathers for weeks.

## 5. Conclusions

Despite the presence of a strong local T cell-mediated melanocyte-specific inflammatory response, Smyth chickens developing vitiligo do not better control HVT infection, replication, and persistence in feathers than non-vitiliginous parental chickens. They even exhibit higher HVT loads at vitiligo onset. There is therefore a mutual positive effect between HVT infection and vitiligo development.

## Supplementary Materials

The following are available online at https://www.mdpi.com/2076-2607/8/10/1613/s1. Table S1: HVT loads in pigmented and non-pigmented feathers, per chicken and per time as well as vitiligo scores.
