# Supplementary material for: Mardivirus Infection and Persistence in Feathers of a Chicken Model Harboring a Local Autoimmune Response
Source: Microorganisms. 2020 Oct 20;8(10):1613. doi: 10.3390/microorganisms8101613 (PMC7589623; doi:10.3390/microorganisms8101613)
Supplement: Supplementary file 1 [file microorganisms-08-01613-s001.pdf]

Table S1. HVT loads in pigmented and non-pigmented feathers, per chicken and per time as well as vitiligo scores.

	Bird Nber	Subject	Chicken Line	Date	Group	vitiligo score	Repigm	HVT Log(A)	HVT Log(B)	HVTLog (A,B)	HVT Log (A, B or (A,B))
Date	1	**B1**	BL	Wk2	BLW2	1		0,000	NA		0,000
Week 2	3	**B2**	BL	Wk2	BLW2	1		0,000	NA		0,000
	8	**B3**	BL	Wk2	BLW2	1		2,567	NA		2,567
	10	**B4**	BL	Wk2	BLW2	1		2,777	NA		2,777
	12	**B5**	BL	Wk2	BLW2	1		2,591	NA		2,591
	15	**B6**	BL	Wk2	BLW2	1		0,000	NA		0,000
	18	**B7**	BL	Wk2	BLW2	1		0,000	NA		0,000
	19	**B8**	BL	Wk2	BLW2	1		2,114	NA		2,114
	20	**B9**	BL	Wk2	BLW2	1		2,714	NA		2,714
	22	**S1**	SL	Wk2	SLW2	1		2,358	NA		2,358
	27	**S2**	SL	Wk2	SLW2	1		0,000	NA		0,000
	36	**S4**	SL	Wk2	SLW2	1		1,786	NA		1,786
	40	**S5**	SL	Wk2	SLW2	1		0,000	NA		0,000
	41	**S6**	SL	Wk2	SLW2	1		2,204	NA		2,204
	42	**S7**	SL	Wk2	SLW2	1		3,140	NA		3,140
	45	**S8**	SL	Wk2	SLW2	1		1,872	NA		1,872
	46	**S9**	SL	Wk2	SLW2	1		1,780	NA		1,780
	49	**S11**	SL	Wk2	SLW2	1		2,079	NA		2,079
	53	**S12**	SL	Wk2	SLW2	1		2,200	NA		2,200
	55	**S13**	SL	Wk2	SLW2	1		3,267	NA		3,267
Date	1	**B1**	BL	Wk5	BLW5	1		1,96	NA		1,96
Week 5	3	**B2**	BL	Wk5	BLW5	1		2,299	NA		2,299
	8	**B3**	BL	Wk5	BLW5	1		1,669	NA		1,669
	10	**B4**	BL	Wk5	BLW5	1		3,179	NA		3,179
	12	**B5**	BL	Wk5	BLW5	1		2,895	NA		2,895
	15	**B6**	BL	Wk5	BLW5	1		0,000	NA		0,000
	18	**B7**	BL	Wk5	BLW5	1		3,988	NA		3,988
	19	**B8**	BL	Wk5	BLW5	1		0,000	NA		0,000
	20	**B9**	BL	Wk5	BLW5	1		2,884	NA		2,884
	22	**S1**	SL	Wk5	SLW5	1		2,200	NA		2,200
	27	**S2**	SL	Wk5	SLW5	1		2,350	NA		2,350
	36	**S4**	SL	Wk5	SLW5	1		2,889	NA		2,889
	40	**S5**	SL	Wk5	SLW5	1		0,000	NA		0,000
	41	**S6**	SL	Wk5	SLW5	1		2,701	NA		2,701
	42	**S7**	SL	Wk5	SLW5	1		2,993	NA		2,993
	45	**S8**	SL	Wk5	SLW5	1		2,548	NA		2,548
	46	**S9**	SL	Wk5	SLW5	1		0,000	NA		0,000
	49	**S11**	SL	Wk5	SLW5	1		0,000	NA		0,000
	53	**S12**	SL	Wk5	SLW5	1		2,130	NA		2,130
	55	**S13**	SL	Wk5	SLW5	1		2,609	NA		2,609
Date	1	**B1**	BL	Wk8	BLW8	1		2,185	NA		2,185
Week 8	3	**B2**	BL	Wk8	BLW8	1		2,496	NA		2,496
	8	**B3**	BL	Wk8	BLW8	4	R	2,751	na		2,751
	10	**B4**	BL	Wk8	BLW8	1		2,590	NA		2,590
	12	**B5**	BL	Wk8	BLW8	1		1,710	NA		1,710
	15	**B6**	BL	Wk8	BLW8	1		0,000	NA		0,000
	18	**B7**	BL	Wk8	BLW8	1		1,701	NA		1,701
	19	**B8**	BL	Wk8	BLW8	1		1,800	NA		1,800
	20	**B9**	BL	Wk8	BLW8	1		2,760	NA		2,760
	22	**S1**	SL	Wk8	SLW8	4		3,778	3,389	3,584	3,584
	27	**S2**	SL	Wk8	SLW8	3		2,775	2,930	2,853	2,853
	36	**S4**	SL	Wk8	SLW8	1		3,037	NA		3,037
	40	**S5**	SL	Wk8	SLW8	1		3,283	NA		3,283
	41	**S6**	SL	Wk8	SLW8	1		2,513	NA		2,513
	42	**S7**	SL	Wk8	SLW8	1		2,761	NA		2,761
	45	**S8**	SL	Wk8	SLW8	5		na	3,140		3,140
	46	**S9**	SL	Wk8	SLW8	1		3,079	NA		3,079
	49	**S11**	SL	Wk8	SLW8	1		3,286	NA		3,286
	53	**S12**	SL	Wk8	SLW8	1		3,332	NA		3,332
	55	**S13**	SL	Wk8	SLW8	1		3,041	NA		3,041
Date	1	**B1**	BL	Wk11	BLW11	1		2,852	NA		2,852
week 11	3	**B2**	BL	Wk11	BLW11	1		1,873	NA		1,873
	8	**B3**	BL	Wk11	BLW11	4		3,076	2,665	2,870	2,870
	10	**B4**	BL	Wk11	BLW11	1		2,657	NA		2,657
	12	**B5**	BL	Wk11	BLW11	1		2,511	NA		2,511
	15	**B6**	BL	Wk11	BLW11	1		2,377	NA		2,377
	18	**B7**	BL	Wk11	BLW11	1		0,000	NA		0,000
	19	**B8**	BL	Wk11	BLW11	1		2,629	NA		2,629
	20	**B9**	BL	Wk11	BLW11	1		0,000	NA		0,000
	22	**S1**	SL	Wk11	SLW11	2	R	3,452	na		3,452
	27	**S2**	SL	Wk11	SLW11	3		2,986	3,004	2,995	2,995
	36	**S4**	SL	Wk11	SLW11	5		na	2,560		2,560
	40	**S5**	SL	Wk11	SLW11	1		2,936	NA		2,936
	41	**S6**	SL	Wk11	SLW11	1		3,255	NA		3,255
	42	**S7**	SL	Wk11	SLW11	1		3,265	NA		3,265
	45	**S8**	SL	Wk11	SLW11	5		na	3,940		3,940
	46	**S9**	SL	Wk11	SLW11	1		2,210	NA		2,210
	49	**S11**	SL	Wk11	SLW11	2	R	3,009	na		3,009
	53	**S12**	SL	Wk11	SLW11	1		1,885	NA		1,885
	55	**S13**	SL	Wk11	SLW11	5		na	2,775		2,775
Date	1	**B1**	BL	Wk14	BLW14	1		2,786	NA		2,786
week 14	3	**B2**	BL	Wk14	BLW14	1		3,410	NA		3,410
	8	**B3**	BL	Wk14	BLW14	4	R	3,210	na		3,210
	10	**B4**	BL	Wk14	BLW14	1		2,863	NA		2,863
	12	**B5**	BL	Wk14	BLW14	1		2,782	NA		2,782
	15	**B6**	BL	Wk14	BLW14	1		0,000	NA		0,000

	18	**B7**	BL	Wk14	BLW14	1		0,000	NA		0,000
	19	**B8**	BL	Wk14	BLW14	1		2,994	NA		2,994
	20	**B9**	BL	Wk14	BLW14	1		4,017	NA		4,017
	22	**S1**	SL	Wk14	SLW14	2	R	3,270	na		3,270
	27	**S2**	SL	Wk14	SLW14	4		1,929	2,974	2,451	2,451
	36	**S4**	SL	Wk14	SLW14	4		2,446	2,665	2,555	2,555
	40	**S5**	SL	Wk14	SLW14	2	R	3,336	na		3,336
	41	**S6**	SL	Wk14	SLW14	4		3,190	2,946	3,068	3,068
	42	**S7**	SL	Wk14	SLW14	1		3,951	NA		3,951
	45	**S8**	SL	Wk14	SLW14	5		na	2,931		2,931
	46	**S9**	SL	Wk14	SLW14	1		2,985	NA		2,985
	49	**S11**	SL	Wk14	SLW14	5		na	2,504		2,504
	53	**S12**	SL	Wk14	SLW14	1		2,714	NA		2,714
	55	**S13**	SL	Wk14	SLW14	5		na	1,794		1,794
Date	1	**B1**	BL	Wk17	BLW17	1		2,415	NA		2,415
week 17	3	**B2**	BL	Wk17	BLW17	1		3,152	NA		3,152
	8	**B3**	BL	Wk17	BLW17	2	R	3,455	na		3,455
	10	**B4**	BL	Wk17	BLW17	1		4,384	NA		4,384
	12	**B5**	BL	Wk17	BLW17	1		3,722	NA		3,722
	15	**B6**	BL	Wk17	BLW17	1		2,455	NA		2,455
	18	**B7**	BL	Wk17	BLW17	1		2,083	NA		2,083
	19	**B8**	BL	Wk17	BLW17	1		3,114	NA		3,114
	20	**B9**	BL	Wk17	BLW17	1		3,207	NA		3,207
	22	**S1**	SL	Wk17	SLW17	2	R	3,568	na		3,568
	27	**S2**	SL	Wk17	SLW17	5		na	3,143		3,143
	36	**S4**	SL	Wk17	SLW17	5		na	2,364		2,364
	40	**S5**	SL	Wk17	SLW17	5		na	3,155		3,155
	41	**S6**	SL	Wk17	SLW17	5		na	3,644		3,644
	42	**S7**	SL	Wk17	SLW17	5		na	3,255		3,255
	45	**S8**	SL	Wk17	SLW17	5		na	2,633		2,633
	46	**S9**	SL	Wk17	SLW17	3		3,127	2,992	3,059	3,059
	49	**S11**	SL	Wk17	SLW17	5		na	2,645		2,645
	53	**S12**	SL	Wk17	SLW17	1		2,912	NA		2,912
	55	**S13**	SL	Wk17	SLW17	5		na	2,829		2,829
Date	1	**B1**	BL	Wk20	BLW20	1		1,455	NA		1,455
Week 20	3	**B2**	BL	Wk20	BLW20	1		2,821	NA		2,821
	8	**B3**	BL	Wk20	BLW20	5		na	3,241		3,241
	10	**B4**	BL	Wk20	BLW20	1		3,009	NA		3,009
	12	**B5**	BL	Wk20	BLW20	1		3,057	NA		3,057
	15	**B6**	BL	Wk20	BLW20	1		2,734	NA		2,734
	18	**B7**	BL	Wk20	BLW20	1		0,000	NA		0,000
	19	**B8**	BL	Wk20	BLW20	1		3,167	NA		3,167
	20	**B9**	BL	Wk20	BLW20	1		3,354	NA		3,354
	22	**S1**	SL	Wk20	SLW20	5		na	2,799		2,799
	27	**S2**	SL	Wk20	SLW20	5		na	2,970		2,970
	36	**S4**	SL	Wk20	SLW20	5		na	0,000		0,000
	40	**S5**	SL	Wk20	SLW20	5		na	3,798		3,798
	41	**S6**	SL	Wk20	SLW20	5		na	2,447		2,447
	42	**S7**	SL	Wk20	SLW20	5		na	4,041		4,041
	45	**S8**	SL	Wk20	SLW20	5		na	2,951		2,951
	46	**S9**	SL	Wk20	SLW20	5		na	3,201		3,201
	49	**S11**	SL	Wk20	SLW20	5		na	2,787		2,787
	53	**S12**	SL	Wk20	SLW20	4		2,958	3,185	3,071	3,071
	55	**S13**	SL	Wk20	SLW20	5		na	3,316		3,316

If birds has vitiligo: sample A is pigmented; sample B is not pigmented; score of 1 = no vitiligo; score of 5 = complete vitiligo
R = repigmentation - i.e., bird had vitiligo episode but all new growth is pigmented; in birds with a score of 4, often times the A sample is not fully pigmented, but not white like the B sample

NA, not developed vitiligo, yet
na, only one type of feathers (either pigmented of not) after vitiligo devlpt
HVTLog (A,B), means of the 2 values in feathers A and B when available
Missing value which was reimputed
